# Dual-type quantile regression approach for mean estimation incorporating sampled and non-sampled data

**DOI:** 10.1016/j.heliyon.2024.e31034

**Published:** 2024-05-14

**Authors:** Abdullah Mohammed Alomair, Soofia Iftikhar

**Affiliations:** a Department of Quantitative Methods, School of Business, King Faisal University, Al-Ahsa 31982, Saudi Arabia; b Department of Statistics, Shaheed Benazir Bhutto Women University Peshawar, KP, Pakistan

**Keywords:** Quantile regression, Sensitive variable, Auxiliary information, Dual-type estimators, SRS

## Abstract

Drawing inspiration from recent advancements in robust mean estimation within finite sampling theory, we introduce a novel dual-type class of mean estimators in a design-based framework. The dual-type class is based on quantile regression and is specifically designed to be effective in the presence of extreme observations. Significantly, it integrates the averages of both sampled observations and non-sampled observations of auxiliary variable. In the initial discussion of this class, it is presumed that the target variable is non-sensitive, signifying its relevance to subjects that respondents do not consider embarrassing when queried directly. In this standard setting, we present specific estimators within the class and determine their theoretical properties. The class's scope broadens to include scenarios where the target variable incorporates sensitive topics, giving rise to nonresponse rates and inaccurate reporting. To alleviate these errors, one can promote respondent cooperation by employing scrambled response methods that obscure the actual value of the sensitive variable. Accordingly, the article delves into discussions on additive methods. Subsequently, a numerical study is conducted using asymmetric data to evaluate the effectiveness of the dual-type class by comparing it with several existing estimators, both in the absence and presence of scrambled responses.

## 1 Introduction

In the realm of sample surveys, a critical and fundamental goal is to enhance the precision of estimating the study variable. To achieve this aim, statisticians have, in recent decades, integrated auxiliary information, acknowledging its capacity to enhance precision. In cases where the correlation coefficients between the study and auxiliary variables exhibit positivity, ratio estimators come into play. Conversely, when a negative correlation is evident, the product estimator is employed to gauge the population mean, Bhushan et al. [1]. Both ratio and product type estimators leverage supplementary information to yield precise and efficient results. The refinement of estimator efficiency is further accomplished by leveraging information pertaining to the characteristics of auxiliary variables.

Enhancing the accuracy and efficiency of estimators for calculating sums, means, and variances etc in finite populations is a standard practice through the incorporation of auxiliary information, which is based on ratio, multiplication, regression, and difference techniques. These estimators offer a notable advantage, leveraging the linear relationship (*ρ*) between the auxiliary and study variables. In certain situations, they yield estimations that are more finely tuned, exhibiting smaller variance compared to estimators relying solely on the arithmetic mean. Among these, the ratio technique distinguishes itself as one of the most commonly applied techniques for estimating the means, sums, and variances of a finite population, particularly when positive correlation coefficients exist between the two variables, Bhushan et al. [2]. The literature boasts numerous studies dedicated to exploring and refining ratio-type estimators. For instance Koyuncu [3] developed class of exponential type estimators. Abid et al. [4], [5] used non-conventional measures of location. For more about efficient estimation of parameter, interested readers may refer to Naz et al. [6]. It is worthy to note that many of these mentioned estimators use ordinary least square regression coefficient.

The OLS method is extensively used in regression analysis. However, the presence of outliers in the data can substantially compromise the accuracy of inferences drawn from it. Selected for its computational simplicity in predicting function parameters, OLS stands out as a famous method. The core aim of OLS is to minimize the sum of squared differences between the actual and expected values derived from a regression model. However, a notable drawback of OLS is its vulnerability to outliers. The presence of a single outlier can markedly impact the regression line, leading to unreliable parameter estimates. In spite of its susceptibility to outliers, OLS continues to be a valuable technique under the condition that the underlying assumptions are satisfied. These assumptions include homoscedasticity, homogeneity, independence of errors, and linearity. In the presence of these conditions, OLS provides the most accurate estimates under optimal circumstances. Due to its user-friendly nature, OLS is frequently employed in analysis, particularly when the specified assumptions are met to a considerable degree. In situations where outliers exist or assumptions are violated, alternative regression methods like weighted or robust regression may be more suitable to guarantee the resilience and accuracy of the analysis Zaman and Bulut [7]. Encounters with outliers or deviations from assumptions are not unusual in real-world data, leading to the endorsement of robust techniques as a preferred solution to tackle these challenges.

Several robust regression techniques, such as LAD, LTS, Huber-M, and Tukey-M, are widely recognized. Robust mean estimation has significantly progressed over the years through the application of these techniques. In the early stages, Zaman and Bulut [8], [9] and Subzar et al. [10] examined how various robust regression methods could be applied to estimate the mean of a finite population based on ratios, especially in the presence of outliers. In a thorough analysis, Begashaw and Yohannes [11] investigated robust regression models for detecting and recognizing outliers. Audu et al. [12] employed robust regression techniques to assess how effective exponential-type estimates are for the population mean. Deville and Sarndal [13] significantly advanced the field by offering dependable calibration approach for calculating the mean parameter through probability sampling.

Recently, Shahzad et al. [14] used robust quantile regression coefficients $q_{\left(0.15,0.25,0.35\right)}$ to estimate the mean in the presence of outliers. These authors introduced a collection of quantile regression based estimators specifically tailored for non-normally distributed data with outliers, with the goal of estimating the population mean. In a related context, Anas et al. [15] defined a similar category of estimators that leverage Linear-moments for estimating the mean parameter in non-normally distributed datasets with outliers under simple random sampling. Shahzad et al. [16] presented a resilient category of quantile based estimators explicitly crafted for estimating population means within the context of stratified random sampling. In a different vein, Rueda and Arcos [17] investigated population quantiles using exponentiation method. Shahzad et al. [18] $q_{\left(0.1,0.2,0.3,0.4,0.5,0.6,0.7,0.8,0.9\right)}$ and Shahzad et al. [19] $q_{\left(0.14,0.24,0.34,0.44,0.54\right)}$ introduced a robust estimation approach for the population mean using quantile regression within the framework of systematic sampling and robust covariance matrices, respectively. Lastly, Yadav and Prasad [20], utilizing robust quantile regression methodologies $q_{\left(0.25,0.35,0.45,0.55,0.65,0.75,0.85\right)}$, examined exponential estimation methods in sampling theory. Koc and Koc [21] extended this work using quantile regression coefficients $q_{\left(0.25,0.50,0.75\right)}$ within a stratified random sampling scheme.

The entire body of literature concerning estimators mentioned in the preceding lines relies on the mean of observations sampled from an auxiliary variable. Srivenkataramana and Tracy [22] proposed that an estimator can be enhanced by incorporating the mean of non-sampled observations of the auxiliary variable. Estimators of this kind are referred to as dual-type estimators. Subsequently, numerous authors have adopted this concept to achieve more efficient estimation, as indicated in the references [23], [24], [25].

To the best of our knowledge, there is no existing study that has employed both quantile regression and the mean of non-sampled observations. Therefore, this study is motivated by this gap and introduces a class of mean estimators based on quantile regression under SRS, incorporating both sampled and non-sampled observations, particularly in the presence of extreme values. Initially, we presume that the variable under investigation is non-sensitive, thus adhering to a conventional estimation framework. Following this, we extend these findings to situations involving a sensitive, deeply personal, potentially threatening, or stigmatized variable. It is assumed that this sensitive variable is observed in sampled units through nonstandard survey techniques, implemented to improve respondent response rate. These methods are linked to the randomized response theory pioneered by Warner [26] and thoroughly demonstrated by Asma et al. [27]. Moreover, a numerical study is conducted to assess the merits of the proposed dual class for the variable of sensitivity. This assessment is conducted using altered observations of the target variable, achieved by randomizing the true observations according to the rules defined in various randomized response models.

The remainder of the paper is structured as follows: In Section 2, we delve into a discussion of reviewed quantile regression based mean estimators. Section 3 introduces our proposed class of dual-type mean estimators, utilizing both sampled and non-sampled observations, along with illustrations of potential members within the class. Transitioning to Section 4, we delve into challenges that might emerge when utilizing direct questioning in surveys, especially concerning sensitive topics. In response, we broaden the proposed class of estimators to accommodate setups involving sensitive information. Section 5 is dedicated to assessing the merits of the dual-type class, utilizing both real and simulated populations. Finally, Section 6 wraps up the article with concluding remarks

## 2 Shahzad et al. [14] estimators

Quantile regression shares similarities with traditional OLS regression as both methods investigate relationships between endogenous and exogenous variables. The key difference lies in the parameter estimation approach: OLS regression selects parameter values minimizing the squared deviation from the regression line, whereas quantile regression selects parameter values minimizing the absolute deviation from the regression line. The later approach is suitable in presence of outliers. Therefore, Shahzad et al. [14] introduced a set of ratio-type mean estimators based on quantile regression, as outlined below:

$${\overset{¯}{y}}_{a_{\left(q\right)}}=\frac{\overset{¯}{y}+{\overset{ˆ}{\alpha }}_{\left(q\right)}(\overset{¯}{X}-\overset{¯}{x})}{\left(F_{1}\overset{¯}{x}+F_{2}\right)}(F_{1}\overset{¯}{X}+F_{2})\;\text{for}\;a=1,2,...,9,$$

with

$${\overset{ˆ}{\alpha }}_{\left(q\right)}=argmin_{\alpha \epsilon R^{p}}\;\rho_{q}(v)\sum_{i=1}^{n}(y_{i}-\langle x_{i},\alpha \rangle ),$$

Here, ${\overset{ˆ}{\alpha }}_{\left(q\right)}$ (coefficient of the quantile regression), $\rho_{q}(v)$ (absolute loss function), for p=2 variables with quantile range qϵ(0,1). For a more in-depth exploration of quantile regression, readers interested in further study are encouraged to consult Koenker and Hallock [28]. Further, let $\left(\overset{¯}{X},\overset{¯}{Y}\right)$ denote the averages of population, and $\left(\overset{¯}{x},\overset{¯}{y}\right)$ represent the averages of sample having size *n*. Additionally, $F_{1}$ and $F_{2}$ can take values from the set (0,1) or may correspond to *X* such as HL (Hodges-Lemon), MR (Mid-Range), TM (Tri-Mean), $C_{x}$ (CV). $v_{i}=\frac{F_{1}\overset{¯}{Y}}{F_{1}\overset{¯}{X}+F_{2}}$ and $\theta =(\frac{1-f}{n})$ for a=1,2,...,9. Further, $S_{y}^{2}$ and $S_{x}^{2}$ are the variances, and $S_{xy}$ be the co-variance. The family members of ${\overset{¯}{y}}_{a_{\left(q\right)}}$ are provided in Table 1. The MSE of ${\overset{¯}{y}}_{a_{\left(q\right)}}$ is

$$\mathrm{MSE}({\overset{¯}{y}}_{a_{\left(q\right)}})=\theta [S_{y}^{2}+v_{i}^{2}S_{x}^{2}+2\alpha_{\left(q\right)}v_{i}S_{x}^{2}+\alpha_{\left(q\right)}^{2}S_{x}^{2}-2v_{i}S_{xy}-2\alpha_{\left(q\right)}S_{xy}]\;\text{for}\;a=1,2,...,9.$$

**Table 1:** Family members of Shahzad et al. [14].

*a*	$\left({\overset{ˆ}{\alpha }}_{\left(q\right)},F_{1},F_{2}\right)$	*a*	$\left({\overset{ˆ}{\alpha }}_{\left(q\right)},F_{1},F_{2}\right)$	*a*	$\left({\overset{ˆ}{\alpha }}_{\left(q\right)},F_{1},F_{2}\right)$
1	$\left({\overset{ˆ}{\alpha }}_{\left(0.15\right)},1,TM\right)$	1	$\left({\overset{ˆ}{\alpha }}_{\left(0.25\right)},1,TM\right)$	1	$\left({\overset{ˆ}{\alpha }}_{\left(0.35\right)},1,TM\right)$
2	$\left({\overset{ˆ}{\alpha }}_{\left(0.15\right)},C_{x},TM\right)$	2	$\left({\overset{ˆ}{\alpha }}_{\left(0.25\right)},C_{x},TM\right)$	2	$\left({\overset{ˆ}{\alpha }}_{\left(0.35\right)},C_{x},TM\right)$
3	$\left({\overset{ˆ}{\alpha }}_{\left(0.15\right)},\rho ,TM\right)$	3	$\left({\overset{ˆ}{\alpha }}_{\left(0.25\right)},\rho ,TM\right)$	3	$\left({\overset{ˆ}{\alpha }}_{\left(0.35\right)},\rho ,TM\right)$
4	$\left({\overset{ˆ}{\alpha }}_{\left(0.15\right)},1,MR\right)$	4	$\left({\overset{ˆ}{\alpha }}_{\left(0.25\right)},1,MR\right)$	4	$\left({\overset{ˆ}{\alpha }}_{\left(0.35\right)},1,MR\right)$
5	$\left({\overset{ˆ}{\alpha }}_{\left(0.15\right)},C_{x},MR\right)$	5	$\left({\overset{ˆ}{\alpha }}_{\left(0.25\right)},C_{x},MR\right)$	5	$\left({\overset{ˆ}{\alpha }}_{\left(0.35\right)},C_{x},MR\right)$
6	$\left({\overset{ˆ}{\alpha }}_{\left(0.15\right)},\rho ,MR\right)$	6	$\left({\overset{ˆ}{\alpha }}_{\left(0.25\right)},\rho ,MR\right)$	6	$\left({\overset{ˆ}{\alpha }}_{\left(0.35\right)},\rho ,MR\right)$
7	$\left({\overset{ˆ}{\alpha }}_{\left(0.15\right)},1,HL\right)$	7	$\left({\overset{ˆ}{\alpha }}_{\left(0.25\right)},1,HL\right)$	7	$\left({\overset{ˆ}{\alpha }}_{\left(0.35\right)},1,HL\right)$
8	$\left({\overset{ˆ}{\alpha }}_{\left(0.15\right)},C_{x},HL\right)$	8	$\left({\overset{ˆ}{\alpha }}_{\left(0.25\right)},C_{x},HL\right)$	8	$\left({\overset{ˆ}{\alpha }}_{\left(0.35\right)},C_{x},HL\right)$
9	$\left({\overset{ˆ}{\alpha }}_{\left(0.15\right)},\rho ,HL\right)$	9	$\left({\overset{ˆ}{\alpha }}_{\left(0.25\right)},\rho ,HL\right)$	9	$\left({\overset{ˆ}{\alpha }}_{\left(0.35\right)},\rho ,HL\right)$

It is important to note that in their article, they employ quantiles $q^{15th}$, $q^{25th}$, and $q^{35th}$ for mean estimation. The numerical study reveals that use of these specified quantiles based regression coefficients significantly improves the efficiency of their defined estimators. It is noteworthy that the defined class, utilizing these three quantiles, comprises twenty-seven members. For clarity, we present the 27 members of the Shahzad et al. [14] class along with their MSE in a concise format as follows:

$${\overset{¯}{y}}_{a_{\left(q\right)}}=\left\{ \begin{array}{cc} \frac{\overset{¯}{y}+{\overset{ˆ}{\alpha }}_{\left(0.15\right)}(\overset{¯}{X}-\overset{¯}{x})}{\left(F_{1}\overset{¯}{x}+F_{2}\right)}(F_{1}\overset{¯}{X}+F_{2})\; & \text{for }(q)=(0.15)=1,2,...,9\\ \;\\ \frac{\overset{¯}{y}+{\overset{ˆ}{\alpha }}_{\left(0.25\right)}(\overset{¯}{X}-\overset{¯}{x})}{\left(F_{1}\overset{¯}{x}+F_{2}\right)}(F_{1}\overset{¯}{X}+F_{2})\; & \text{for }(q)=(0.25)=1,2,...,9\\ \;\\ \frac{\overset{¯}{y}+{\overset{ˆ}{\alpha }}_{\left(0.35\right)}(\overset{¯}{X}-\overset{¯}{x})}{\left(F_{1}\overset{¯}{x}+F_{2}\right)}(F_{1}\overset{¯}{X}+F_{2})\; & \text{for }(q)=(0.35)=1,2,...,9\text{,} \end{array}\right.$$

$$\mathrm{MSE}({\overset{¯}{y}}_{a_{\left(q\right)}})=\left\{ \begin{array}{cc} \theta [S_{y}^{2}+v_{i}^{2}S_{x}^{2}+2\alpha_{\left(0.15\right)}v_{i}S_{x}^{2}+\alpha_{\left(0.15\right)}^{2}S_{x}^{2}-2v_{i}S_{xy}-2\alpha_{\left(0.15\right)}S_{xy}]\; & \\ \;\\ \theta [S_{y}^{2}+v_{i}^{2}S_{x}^{2}+2\alpha_{\left(0.25\right)}v_{i}S_{x}^{2}+\alpha_{\left(0.25\right)}^{2}S_{x}^{2}-2v_{i}S_{xy}-2\alpha_{\left(0.25\right)}S_{xy}]\; & \\ \;\\ \theta [S_{y}^{2}+v_{i}^{2}S_{x}^{2}+2\alpha_{\left(0.35\right)}v_{i}S_{x}^{2}+\alpha_{\left(0.35\right)}^{2}S_{x}^{2}-2v_{i}S_{xy}-2\alpha_{\left(0.35\right)}S_{xy}]\; & \end{array}\right.$$

## 3 Proposed estimators using sampled and non-samples auxiliary information

Outliers refer to observations in a dataset that appear inconsistent with the remaining data. The presence of outliers can significantly impact mean estimation, a crucial measure of central tendency. Mean estimators relying on OLS regression coefficients are often considered optimal for population mean estimation. However, outliers can exert a substantial influence on the traditional OLS regression coefficient, leading to potential inaccuracies in the population mean estimate. To address this issue, quantile regression, as suggested by Shahzad et al. [14], can be employed. However, existing estimators based on this approach only consider the mean of sampled observations from an auxiliary variable. Drawing inspiration from Srivenkataramana and Tracy [22], who proposed that incorporating the mean of non-sampled observations enhances estimators, and building upon the works of Searls [29] and Shahzad et al. [14], we introduce a class of mean estimators based on quantile regression, as follows:

(1) $${\overset{¯}{y}}_{t_{\left(q\right)}}=k_{\left(q\right)}\frac{\overset{¯}{y}}{\overset{¯}{X}}{\overset{¯}{x}}^{⁎}+{\overset{ˆ}{\alpha }}_{\left(q\right)}(\overset{¯}{X}-\overset{¯}{x})\;for\;q=0.15,0.25,0.35.$$

This class utilizes both the mean of sampled observations and the mean of non-sampled observations from the auxiliary variable. Note that, above mentioned expressions of proposed estimators are in generalized form. So, we can write ${\overset{¯}{y}}_{t_{\left(q\right)}}$ for (q)=0.15,0.25,0.35 as given below:

$${\overset{¯}{y}}_{t_{\left(q\right)}}=\left\{ \begin{array}{cc} k_{\left(0.15\right)}\frac{\overset{¯}{y}}{\overset{¯}{X}}{\overset{¯}{x}}^{⁎}+{\overset{ˆ}{\alpha }}_{\left(0.15\right)}(\overset{¯}{X}-\overset{¯}{x})\; & \text{for }(q)=(0.15)\\ \;\\ k_{\left(0.25\right)}\frac{\overset{¯}{y}}{\overset{¯}{X}}{\overset{¯}{x}}^{⁎}+{\overset{ˆ}{\alpha }}_{\left(0.25\right)}(\overset{¯}{X}-\overset{¯}{x})\; & \text{for }(q)=(0.25)\\ \;\\ k_{\left(0.35\right)}\frac{\overset{¯}{y}}{\overset{¯}{X}}{\overset{¯}{x}}^{⁎}+{\overset{ˆ}{\alpha }}_{\left(0.35\right)}(\overset{¯}{X}-\overset{¯}{x})\; & \text{for }(q)=(0.35) \end{array}\right.$$

where ${\overset{¯}{x}}^{⁎}=\frac{N\overset{¯}{X}-n\overset{¯}{x}}{N-n}=\frac{1}{N-n}\sum_{i=1}^{N-n}x_{i}$ is representing the mean of non-sampled units of X.

The MSE of proposed family of estimators is given below

$$\mathrm{MSE}({\overset{¯}{y}}_{t_{\left(q\right)}})=\theta \left[k_{\left(q\right)}^{2}A+D_{\left(q\right)}-2k_{\left(q\right)}C_{\left(q\right)}\right]$$

where

$$A={\overset{¯}{Y}}^{2}\left\{C_{y}^{2}+{\left(\frac{n}{N-n}\right)}^{2}C_{x}^{2}-2\left(\frac{n}{N-n}\right)\rho_{xy}C_{y}C_{x}\right\},\;D_{\left(q\right)}=\alpha_{\left(q\right)}^{2}{\overset{¯}{X}}^{2}C_{x}^{2},$$

and

$$C_{\left(q\right)}=\alpha_{\left(q\right)}\overset{¯}{X}\overset{¯}{Y}\left\{\rho_{xy}C_{y}C_{x}+\left(\frac{n}{N-n}\right)C_{x}^{2}\right\}.$$

Now, differentiating w.r.t. $k_{\left(q\right)}$ and equating to zero:

$$k_{\left(q\right)}^{opt}=\frac{C_{\left(q\right)}}{A}.$$

Hence, minimum MSE of ${\overset{¯}{y}}_{t_{\left(q\right)}}$ is

$$\mathrm{MSE}{\left({\overset{¯}{y}}_{t_{\left(q\right)}}\right)}_{min}=\theta \left[D_{\left(q\right)}-\frac{C_{\left(q\right)}^{2}}{A}\right].$$

Utilizing three referenced quantiles, let us present ${\mathrm{MSE}}_{min}$ of ${\overset{¯}{y}}_{t_{\left(q\right)}}$ as follows

$${\mathrm{MSE}}_{min}({\overset{¯}{y}}_{t_{\left(q\right)}})=\left\{ \begin{array}{cc} \theta \left[D_{\left(0.15\right)}-\frac{C_{\left(0.15\right)}^{2}}{A}\right]\; & \text{for }(q)=(0.15)\\ \;\\ \theta \left[D_{\left(0.25\right)}-\frac{C_{\left(0.25\right)}^{2}}{A}\right]\; & \text{for }(q)=(0.25)\\ \;\\ \theta \left[D_{\left(0.35\right)}-\frac{C_{\left(0.35\right)}^{2}}{A}\right]\; & \text{for }(q)=(0.35) \end{array}\right.$$

## 4 Sensitive research

Questions pertaining to miscarriage, rash driving, intolerance, betting, drunkenness, drug addiction, and erotic behavior are widely acknowledged as sensitive (delicate), as they may be perceived as intrusive into the respondent's personal space. Direct questions concerning private, stigmatizing, or perhaps damning things are sometimes met with erroneous or noncommittal replies when research is being done on sensitive themes. Nonresponses and the provision of untruthful information contribute to nonsampling errors that are challenging to address, significantly compromising the data quality and subsequent analyses. This involves drawing conclusions about undisclosed traits of the population under examination. To address these errors, primarily associated with socially desirable bias, survey statisticians have developed various strategies to improve respondent cooperation while still maintaining privacy. One method for enhancing reporting on sensitive topics is to reduce the impact of the interviewer. This goal is often accomplished using telephonic interviews or self-administered questionnaires completed with paper and pencil. Alternatively, indirect techniques of questioning may be utilized.

The basis for adopting this unconventional data-collection method lies in avoiding direct questioning of survey participants. This approach encompasses diverse strategies designed to extract sensitive information. In this context, specifically when the target variable is considered sensitive and there is a need to estimate its population mean, our attention will be directed towards the randomized response approach, developed by Warner [26]. The methodology, initially created to deal with binary variables and calculate the proportion of people who have a stigmatizing characteristic, the approach has rapidly been extended to include sensitive quantitative factors related to other facets of life. These include metrics like the count of extramarital partnerships, the number of unintended pregnancies [30], scenarios of tax fraud and per week of undocumented work [31], the number of cannabis cigarettes smoked [32], and the frequency with which students encountering challenges in controlling unconventional sexual activities [33].

Several strategies have been proposed in the literature to protect respondents' anonymity and obfuscate responses. The concept involves urging respondents to withhold the actual response of the delicate variable. Instead, they are instructed to disclose a modified value achieved by algebraically perturbing the true response using independent scrambling variables. The researcher is well aware of the distributions of these variables, which have no relationship whatsoever to the sensitive variable or to each other. In this paper, we adapt the additive model proposed by Pollock and Bek [34], denoted as Z=Y+U. Here, *Z* represents the sensitive variable, and *U* signifies the scrambled response.

Advancements in the randomized response approach have facilitated the integration of auxiliary information for efficient estimation. In this context, Diana and Perri [35] presented a versatile scrambling mechanism that allows for the utilization of a supplementary variable, whether it is scrambled or unscrambled.

For the scope of this study, we consider the scenario where the *X* remains unscrambled. Consequently, we adapt the proposed class, as defined in equation (1), to the situation where *Y* is assumed to be sensitive, and data collection follows the Pollock and Bek [34] method mentioned earlier. The set of estimators with sensitive responses can be easily derived from equation (1) by substituting *y* with *z* as given below:

$${\overset{¯}{z}}_{t_{\left(q\right)}}=\left\{ \begin{array}{cc} k_{\left(0.15\right)}\frac{\overset{¯}{z}}{\overset{¯}{X}}{\overset{¯}{x}}^{⁎}+{\overset{ˆ}{\alpha }}_{\left(0.15\right)}(\overset{¯}{X}-\overset{¯}{x})\; & \text{for }(q)=(0.15)\\ \;\\ k_{\left(0.25\right)}\frac{\overset{¯}{z}}{\overset{¯}{X}}{\overset{¯}{x}}^{⁎}+{\overset{ˆ}{\alpha }}_{\left(0.25\right)}(\overset{¯}{X}-\overset{¯}{x})\; & \text{for }(q)=(0.25)\\ \;\\ k_{\left(0.35\right)}\frac{\overset{¯}{z}}{\overset{¯}{X}}{\overset{¯}{x}}^{⁎}+{\overset{ˆ}{\alpha }}_{\left(0.35\right)}(\overset{¯}{X}-\overset{¯}{x})\; & \text{for }(q)=(0.35) \end{array}\right.$$

The formulas given in Section 3 for the nonsensitive scenario may be used to determine the minimal MSE by replacing variable *Y* with *Z*. Therefore, we obtain:

$${\mathrm{MSE}}_{min}({\overset{¯}{z}}_{t_{\left(q\right)}})=\left\{ \begin{array}{cc} \theta \left[D_{z(0.15)}-\frac{C_{z(0.15)}^{2}}{A_{z}}\right]\; & \text{for }(q)=(0.15)\\ \;\\ \theta \left[D_{z(0.25)}-\frac{C_{z(0.25)}^{2}}{A_{z}}\right]\; & \text{for }(q)=(0.25)\\ \;\\ \theta \left[D_{z(0.35)}-\frac{C_{z(0.35)}^{2}}{A_{z}}\right]\; & \text{for }(q)=(0.35) \end{array}\right.$$

where

$$A_{z}={\overset{¯}{Z}}^{2}\left\{C_{z}^{2}+{\left(\frac{n}{N-n}\right)}^{2}C_{x}^{2}-2\left(\frac{n}{N-n}\right)\rho_{xz}C_{z}C_{x}\right\},\;D_{z(q)}=\alpha_{\left(q\right)}^{2}{\overset{¯}{X}}^{2}C_{x}^{2},$$

and

$$C_{z(q)}=\alpha_{\left(q\right)}\overset{¯}{X}\overset{¯}{Z}\left\{\rho_{xz}C_{z}C_{x}+\left(\frac{n}{N-n}\right)C_{z}^{2}\right\}.$$

We refrain from providing the specifics of the modified expressions of existing estimators as they can be readily deduced without requiring any extra effort.

## 5 Numerical illustration

In this segment, we are evaluating the effectiveness of both proposed and existing estimators by employing three datasets as outlined below.

### 5.1 COVID-19 (population-1)

Coronaviruses form a varied category of viruses. The onset of the coronavirus disease (COVID-19), which initially originated in Wuhan, the capital city of Hubei Province in China, emerged at the end of 2019 and was later categorized as a pandemic by the World Health Organization (WHO). As of March 23, 2020, The focal point of the COVID-19 outbreak had moved to Europe and the Middle East, as the situation in China had largely stabilized.

These viruses, responsible for a spectrum of respiratory ailments in humans, range from mild colds and flu to more severe conditions. The global count of confirmed COVID-19 cases is escalating rapidly across numerous countries. Since the close of December 2019, the coronavirus has claimed the lives of 1,557,000 people worldwide and infected over 68 million individuals. The pandemic has also led to prompting the suspension of various activities due to the widespread fear of the disease resurging, particularly during the winter months.

For our numerical analysis, we focus on COVID-19 pandemic data for the European continent, obtained from January 22, 2020, to August 23, 2020, sourced from (https://www.worldometers.info/coronavirus, accessed on 10 September 2020), and approval was not needed. The data includes information on: *X*: Total number of cases, *Y*: Total number of recoveries. Further, N=48 and n=15.

### 5.2 US-cereals (population-2)

We employ the “US-cereals” dataset, offering details on 65 frequently found breakfast cereals in the United States. This dataset is extracted from the obligatory food labels displayed on the cereal packaging. The measurements have been standardized to a serving size equivalent to one American cup. This dataset is sourced from the ASA Statistical Graphics Exposition and has been previously employed by Venables and Ripley [36].

Given that the dataset comprises multiple variables, we designate, *X*=grams of fiber in one portion, while *Y*=grams of potassium. Further, N=65 and n=20.

### 5.3 Simulation (population-3)

For empirical study, we use simulated population of size 1000. Where random variables $X_{e}$ and $Y_{e}$ generated as follows:

$$Y_{e}=\delta_{e}+K_{e}X_{e}+\varepsilon_{e}X_{e}^{q_{e}}$$

where $X_{e}$ follows Gamma $\left(A_{e},B_{e}\right)$. $A_{e}=2.0$ and $B_{e}=3.1]$. $\varepsilon_{e}$ follows standard Gaussian. Further, $\delta_{e}=5$, $q_{e}=1.6$ and $K_{e}=2$.

Following Shahzad et al. [14], 1000 samples of n=50 were selected under the SRS design. After that, the mean estimate $\left({\overset{ˆ}{\phi }}_{e}^{\left(h_{1}\right)},{\overset{ˆ}{\phi }}_{e}^{\left(h_{2}\right)}\right)$ of study variable was calculated. Note that ${\overset{ˆ}{\phi }}_{e}^{\left(h_{1}\right)}$ is representing reviewed estimators and ${\overset{ˆ}{\phi }}_{e}^{\left(h_{2}\right)}$ is representing proposed estimators. The MSE were calculated. The PREs calculated based on MSE as given below:

$$\text{PRE}=\frac{\text{MSE}({\overset{ˆ}{\phi }}_{e}^{\left(h_{1}\right)})}{\text{MSE}({\overset{ˆ}{\phi }}_{e}^{\left(h_{2}\right)})}\times 100$$

The visual representations of the considered populations reveal the presence of outliers, making them conducive for the application of unconventional location measures, as mentioned by Shahzad et al. [14]. Additionally, Shahzad et al. [14] plots are well-suited for quantile regression analysis. Further, the PRE results are provided in Table 2, Table 3, Table 4.

**Table 2:** PRE based performance assessment of ${\overset{¯}{y}}_{t_{\left(q\right)}}$ vs ${\overset{¯}{y}}_{a_{\left(q\right)}}$ using COVID-19 data.

Estimators	${\overset{¯}{y}}_{t_{\left(0.15\right)}}$	${\overset{¯}{y}}_{t_{\left(0.25\right)}}$	${\overset{¯}{y}}_{t_{\left(0.35\right)}}$
${\overset{¯}{y}}_{1_{\left(0.15\right)}}$	2723.126	586.277	601.197
${\overset{¯}{y}}_{2_{\left(0.15\right)}}$	2784.940	599.586	614.844
${\overset{¯}{y}}_{3_{\left(0.15\right)}}$	2717.526	585.072	599.961
${\overset{¯}{y}}_{4_{\left(0.15\right)}}$	3588.658	772.623	792.285
${\overset{¯}{y}}_{5_{\left(0.15\right)}}$	3279.657	706.096	724.065
${\overset{¯}{y}}_{6_{\left(0.15\right)}}$	3753.073	808.021	828.583
${\overset{¯}{y}}_{7_{\left(0.15\right)}}$	2716.370	584.823	599.705
${\overset{¯}{y}}_{8_{\left(0.15\right)}}$	2765.433	595.386	610.537
${\overset{¯}{y}}_{9_{\left(0.15\right)}}$	2727.545	587.229	602.173
${\overset{¯}{y}}_{1_{\left(0.25\right)}}$	2958.034	636.852	653.059
${\overset{¯}{y}}_{2_{\left(0.25\right)}}$	3161.411	680.638	697.959
${\overset{¯}{y}}_{3_{\left(0.25\right)}}$	2842.109	611.894	627.465
${\overset{¯}{y}}_{4_{\left(0.25\right)}}$	2996.791	645.196	661.615
${\overset{¯}{y}}_{5_{\left(0.25\right)}}$	2835.873	610.551	626.089
${\overset{¯}{y}}_{6_{\left(0.25\right)}}$	3093.201	665.953	682.900
${\overset{¯}{y}}_{7_{\left(0.25\right)}}$	2906.665	625.792	641.718
${\overset{¯}{y}}_{8_{\left(0.25\right)}}$	3109.611	669.486	686.523
${\overset{¯}{y}}_{9_{\left(0.25\right)}}$	2801.117	603.068	618.415
${\overset{¯}{y}}_{1_{\left(0.35\right)}}$	2948.849	634.875	651.031
${\overset{¯}{y}}_{2_{\left(0.35\right)}}$	3148.928	677.951	695.203
${\overset{¯}{y}}_{3_{\left(0.35\right)}}$	2835.495	610.470	626.005
${\overset{¯}{y}}_{4_{\left(0.35\right)}}$	3006.865	647.365	663.839
${\overset{¯}{y}}_{5_{\left(0.35\right)}}$	2842.499	611.978	627.551
${\overset{¯}{y}}_{6_{\left(0.35\right)}}$	3104.860	668.463	685.474
${\overset{¯}{y}}_{7_{\left(0.35\right)}}$	2898.519	624.039	639.919
${\overset{¯}{y}}_{8_{\left(0.35\right)}}$	3097.880	666.960	683.933
${\overset{¯}{y}}_{9_{\left(0.35\right)}}$	2795.691	601.900	617.217

**Table 3:** PRE based performance assessment of ${\overset{¯}{y}}_{t_{\left(q\right)}}$ vs ${\overset{¯}{y}}_{a_{\left(q\right)}}$ using US-cereals data.

Estimators	${\overset{¯}{y}}_{t_{\left(0.15\right)}}$	${\overset{¯}{y}}_{t_{\left(0.25\right)}}$	${\overset{¯}{y}}_{t_{\left(0.35\right)}}$
${\overset{¯}{y}}_{1_{\left(0.15\right)}}$	596.222	777.145	940.640
${\overset{¯}{y}}_{2_{\left(0.15\right)}}$	494.722	644.846	780.507
${\overset{¯}{y}}_{3_{\left(0.15\right)}}$	490.003	638.694	773.062
${\overset{¯}{y}}_{4_{\left(0.15\right)}}$	504.762	657.932	796.347
${\overset{¯}{y}}_{5_{\left(0.15\right)}}$	446.625	582.154	704.627
${\overset{¯}{y}}_{6_{\left(0.15\right)}}$	444.357	579.197	701.048
${\overset{¯}{y}}_{7_{\left(0.15\right)}}$	594.764	775.245	938.340
${\overset{¯}{y}}_{8_{\left(0.15\right)}}$	493.850	643.709	779.132
${\overset{¯}{y}}_{9_{\left(0.15\right)}}$	489.168	637.606	771.745
${\overset{¯}{y}}_{1_{\left(0.25\right)}}$	564.745	736.118	890.981
${\overset{¯}{y}}_{2_{\left(0.25\right)}}$	474.763	618.829	749.018
${\overset{¯}{y}}_{3_{\left(0.25\right)}}$	470.733	613.577	742.660
${\overset{¯}{y}}_{4_{\left(0.25\right)}}$	483.404	630.092	762.650
${\overset{¯}{y}}_{5_{\left(0.25\right)}}$	435.329	567.430	686.805
${\overset{¯}{y}}_{6_{\left(0.25\right)}}$	433.619	565.201	684.107
${\overset{¯}{y}}_{7_{\left(0.25\right)}}$	563.425	734.396	888.897
${\overset{¯}{y}}_{8_{\left(0.25\right)}}$	474.016	617.857	747.840
${\overset{¯}{y}}_{9_{\left(0.25\right)}}$	470.023	612.651	741.540
${\overset{¯}{y}}_{1_{\left(0.35\right)}}$	546.151	711.880	861.645
${\overset{¯}{y}}_{2_{\left(0.35\right)}}$	463.569	604.239	731.358
${\overset{¯}{y}}_{3_{\left(0.35\right)}}$	459.983	599.565	725.700
${\overset{¯}{y}}_{4_{\left(0.35\right)}}$	471.311	614.330	743.572
${\overset{¯}{y}}_{5_{\left(0.35\right)}}$	429.703	560.096	677.928
${\overset{¯}{y}}_{6_{\left(0.35\right)}}$	428.352	558.335	675.797
${\overset{¯}{y}}_{7_{\left(0.35\right)}}$	544.918	710.273	859.700
${\overset{¯}{y}}_{8_{\left(0.35\right)}}$	462.904	603.372	730.308
${\overset{¯}{y}}_{9_{\left(0.35\right)}}$	459.353	598.743	724.706

**Table 4:** PRE based performance assessment of ${\overset{¯}{y}}_{t_{\left(q\right)}}$ vs ${\overset{¯}{y}}_{a_{\left(q\right)}}$ using Gamma generated population.

Estimators	${\overset{¯}{y}}_{t_{\left(0.15\right)}}$	${\overset{¯}{y}}_{t_{\left(0.25\right)}}$	${\overset{¯}{y}}_{t_{\left(0.35\right)}}$
${\overset{¯}{y}}_{1_{\left(0.15\right)}}$	3609.500	1850.790	1181.030
${\overset{¯}{y}}_{2_{\left(0.15\right)}}$	2238.121	1147.608	732.314
${\overset{¯}{y}}_{3_{\left(0.15\right)}}$	2501.880	1282.852	818.616
${\overset{¯}{y}}_{4_{\left(0.15\right)}}$	1648.545	845.300	539.405
${\overset{¯}{y}}_{5_{\left(0.15\right)}}$	1075.058	551.241	351.759
${\overset{¯}{y}}_{6_{\left(0.15\right)}}$	1168.064	598.931	382.191
${\overset{¯}{y}}_{7_{\left(0.15\right)}}$	3490.100	1789.567	1141.962
${\overset{¯}{y}}_{8_{\left(0.15\right)}}$	2155.914	1105.456	705.416
${\overset{¯}{y}}_{9_{\left(0.15\right)}}$	2410.810	1236.155	788.818
${\overset{¯}{y}}_{1_{\left(0.25\right)}}$	4152.524	2129.229	1358.708
${\overset{¯}{y}}_{2_{\left(0.25\right)}}$	2633.651	1350.419	861.732
${\overset{¯}{y}}_{3_{\left(0.25\right)}}$	2929.980	1502.363	958.691
${\overset{¯}{y}}_{4_{\left(0.25\right)}}$	1958.146	1004.050	640.706
${\overset{¯}{y}}_{5_{\left(0.25\right)}}$	1262.292	647.247	413.022
${\overset{¯}{y}}_{6_{\left(0.25\right)}}$	1380.616	707.918	451.738
${\overset{¯}{y}}_{7_{\left(0.25\right)}}$	4021.987	2062.295	1315.996
${\overset{¯}{y}}_{8_{\left(0.25\right)}}$	2540.693	1302.754	831.316
${\overset{¯}{y}}_{9_{\left(0.25\right)}}$	2827.970	1450.056	925.313
${\overset{¯}{y}}_{1_{\left(0.35\right)}}$	4674.251	2396.747	1529.417
${\overset{¯}{y}}_{2_{\left(0.35\right)}}$	3024.556	1550.857	989.636
${\overset{¯}{y}}_{3_{\left(0.35\right)}}$	3349.774	1717.614	1096.047
${\overset{¯}{y}}_{4_{\left(0.35\right)}}$	2272.834	1165.407	743.672
${\overset{¯}{y}}_{5_{\left(0.35\right)}}$	1468.445	752.952	480.475
${\overset{¯}{y}}_{6_{\left(0.35\right)}}$	1609.225	825.138	526.539
${\overset{¯}{y}}_{7_{\left(0.35\right)}}$	4533.836	2324.749	1483.473
${\overset{¯}{y}}_{8_{\left(0.35\right)}}$	2922.061	1498.302	956.100
${\overset{¯}{y}}_{9_{\left(0.35\right)}}$	3238.059	1660.332	1059.494

In the sensitive scenario outlined in Section 4, we apply the Pollock and Bek [34] scrambling technique to perturb the values of the target variable. The scrambled variable, denoted as S, is assumed to be normally distributed with a mean of zero, and its standard deviation is set to 10% of *X*. The results for the sensitive case in terms of PRE are presented in Table 5, Table 6, Table 7.

**Table 5:** PRE of estimators for sensitive case using COVID-19 data.

Estimators	${\overset{¯}{y}}_{t_{\left(0.15\right)}}$	${\overset{¯}{y}}_{t_{\left(0.25\right)}}$	${\overset{¯}{y}}_{t_{\left(0.35\right)}}$
${\overset{¯}{y}}_{1_{\left(0.15\right)}}$	2308.230	34581.97	22657.44
${\overset{¯}{y}}_{2_{\left(0.15\right)}}$	2312.300	34642.95	22697.39
${\overset{¯}{y}}_{3_{\left(0.15\right)}}$	2280.386	34164.83	22384.13
${\overset{¯}{y}}_{4_{\left(0.15\right)}}$	2285.598	34242.90	22435.28
${\overset{¯}{y}}_{5_{\left(0.15\right)}}$	2289.508	34301.49	22473.67
${\overset{¯}{y}}_{6_{\left(0.15\right)}}$	2279.040	34144.65	22370.92
${\overset{¯}{y}}_{7_{\left(0.15\right)}}$	2306.959	34562.93	22644.96
${\overset{¯}{y}}_{8_{\left(0.15\right)}}$	2311.366	34628.96	22688.23
${\overset{¯}{y}}_{9_{\left(0.15\right)}}$	2280.187	34161.83	22382.17
${\overset{¯}{y}}_{1_{\left(0.25\right)}}$	2165.452	32442.87	21255.94
${\overset{¯}{y}}_{2_{\left(0.25\right)}}$	2165.269	32440.14	21254.15
${\overset{¯}{y}}_{3_{\left(0.25\right)}}$	2168.487	32488.35	21285.74
${\overset{¯}{y}}_{4_{\left(0.25\right)}}$	2167.657	32475.91	21277.58
${\overset{¯}{y}}_{5_{\left(0.25\right)}}$	2167.119	32467.85	21272.30
${\overset{¯}{y}}_{6_{\left(0.25\right)}}$	2168.724	32491.90	21288.06
${\overset{¯}{y}}_{7_{\left(0.25\right)}}$	2165.521	32443.91	21256.62
${\overset{¯}{y}}_{8_{\left(0.25\right)}}$	2165.306	32440.69	21254.51
${\overset{¯}{y}}_{9_{\left(0.25\right)}}$	2168.522	32488.87	21286.08
${\overset{¯}{y}}_{1_{\left(0.35\right)}}$	2170.259	32514.89	21303.12
${\overset{¯}{y}}_{2_{\left(0.35\right)}}$	2171.057	32526.85	21310.96
${\overset{¯}{y}}_{3_{\left(0.35\right)}}$	2166.171	32453.65	21263.00
${\overset{¯}{y}}_{4_{\left(0.35\right)}}$	2166.734	32462.09	21268.53
${\overset{¯}{y}}_{5_{\left(0.35\right)}}$	2167.223	32469.40	21273.32
${\overset{¯}{y}}_{6_{\left(0.35\right)}}$	2166.043	32451.73	21261.74
${\overset{¯}{y}}_{7_{\left(0.35\right)}}$	2170.019	32511.30	21300.77
${\overset{¯}{y}}_{8_{\left(0.35\right)}}$	2170.870	32524.05	21309.13
${\overset{¯}{y}}_{9_{\left(0.35\right)}}$	2166.152	32453.36	21262.81

**Table 6:** PRE of estimators for sensitive case using US-cereals data.

Estimators	${\overset{¯}{y}}_{t_{\left(0.15\right)}}$	${\overset{¯}{y}}_{t_{\left(0.25\right)}}$	${\overset{¯}{y}}_{t_{\left(0.35\right)}}$
${\overset{¯}{y}}_{1_{\left(0.15\right)}}$	641.542	682.670	965.720
${\overset{¯}{y}}_{2_{\left(0.15\right)}}$	535.425	569.749	805.981
${\overset{¯}{y}}_{3_{\left(0.15\right)}}$	519.064	552.340	781.353
${\overset{¯}{y}}_{4_{\left(0.15\right)}}$	546.144	581.156	822.117
${\overset{¯}{y}}_{5_{\left(0.15\right)}}$	482.382	513.306	726.135
${\overset{¯}{y}}_{6_{\left(0.15\right)}}$	474.042	504.432	713.581
${\overset{¯}{y}}_{7_{\left(0.15\right)}}$	640.043	681.074	963.463
${\overset{¯}{y}}_{8_{\left(0.15\right)}}$	534.490	568.755	804.574
${\overset{¯}{y}}_{9_{\left(0.15\right)}}$	518.252	551.476	780.130
${\overset{¯}{y}}_{1_{\left(0.25\right)}}$	633.013	673.594	952.882
${\overset{¯}{y}}_{2_{\left(0.25\right)}}$	529.668	563.623	797.315
${\overset{¯}{y}}_{3_{\left(0.25\right)}}$	513.862	546.805	773.522
${\overset{¯}{y}}_{4_{\left(0.25\right)}}$	540.051	574.672	812.944
${\overset{¯}{y}}_{5_{\left(0.25\right)}}$	478.710	509.399	720.607
${\overset{¯}{y}}_{6_{\left(0.25\right)}}$	470.815	500.998	708.723
${\overset{¯}{y}}_{7_{\left(0.25\right)}}$	631.547	672.034	950.674
${\overset{¯}{y}}_{8_{\left(0.25\right)}}$	528.763	562.661	795.953
${\overset{¯}{y}}_{9_{\left(0.25\right)}}$	513.079	545.971	772.343
${\overset{¯}{y}}_{1_{\left(0.35\right)}}$	592.862	630.869	892.442
${\overset{¯}{y}}_{2_{\left(0.35\right)}}$	503.502	535.780	757.927
${\overset{¯}{y}}_{3_{\left(0.35\right)}}$	490.496	521.941	738.349
${\overset{¯}{y}}_{4_{\left(0.35\right)}}$	512.186	545.022	771.001
${\overset{¯}{y}}_{5_{\left(0.35\right)}}$	463.065	492.751	697.057
${\overset{¯}{y}}_{6_{\left(0.35\right)}}$	457.415	486.738	688.551
${\overset{¯}{y}}_{7_{\left(0.35\right)}}$	591.562	629.485	890.484
${\overset{¯}{y}}_{8_{\left(0.35\right)}}$	502.750	534.980	756.796
${\overset{¯}{y}}_{9_{\left(0.35\right)}}$	489.859	521.263	737.391

**Table 7:** PRE of estimators for sensitive case using Gamma generated population.

Estimators	${\overset{¯}{y}}_{t_{\left(0.15\right)}}$	${\overset{¯}{y}}_{t_{\left(0.25\right)}}$	${\overset{¯}{y}}_{t_{\left(0.35\right)}}$
${\overset{¯}{y}}_{1_{\left(0.15\right)}}$	3741.985	2161.833	1112.917
${\overset{¯}{y}}_{2_{\left(0.15\right)}}$	2284.267	1319.675	679.371
${\overset{¯}{y}}_{3_{\left(0.15\right)}}$	2713.594	1567.707	807.059
${\overset{¯}{y}}_{4_{\left(0.15\right)}}$	1630.609	942.041	484.965
${\overset{¯}{y}}_{5_{\left(0.15\right)}}$	1197.815	692.006	356.246
${\overset{¯}{y}}_{6_{\left(0.15\right)}}$	1295.663	748.535	385.347
${\overset{¯}{y}}_{7_{\left(0.15\right)}}$	3601.117	2080.450	1071.021
${\overset{¯}{y}}_{8_{\left(0.15\right)}}$	2194.464	1267.793	652.663
${\overset{¯}{y}}_{9_{\left(0.15\right)}}$	2605.437	1505.222	774.892
${\overset{¯}{y}}_{1_{\left(0.25\right)}}$	4178.410	2413.966	1242.715
${\overset{¯}{y}}_{2_{\left(0.25\right)}}$	2581.135	1491.182	767.664
${\overset{¯}{y}}_{3_{\left(0.25\right)}}$	3057.803	1766.565	909.432
${\overset{¯}{y}}_{4_{\left(0.25\right)}}$	1833.570	1059.296	545.328
${\overset{¯}{y}}_{5_{\left(0.25\right)}}$	1289.925	745.220	383.641
${\overset{¯}{y}}_{6_{\left(0.25\right)}}$	1423.022	822.113	423.226
${\overset{¯}{y}}_{7_{\left(0.25\right)}}$	4026.143	2325.998	1197.429
${\overset{¯}{y}}_{8_{\left(0.25\right)}}$	2480.379	1432.973	737.698
${\overset{¯}{y}}_{9_{\left(0.25\right)}}$	2938.388	1697.576	873.916
${\overset{¯}{y}}_{1_{\left(0.35\right)}}$	4967.227	2869.684	1477.320
${\overset{¯}{y}}_{2_{\left(0.35\right)}}$	3140.925	1814.586	934.153
${\overset{¯}{y}}_{3_{\left(0.35\right)}}$	3695.285	2134.853	1099.027
${\overset{¯}{y}}_{4_{\left(0.35\right)}}$	2239.246	1293.665	665.982
${\overset{¯}{y}}_{5_{\left(0.35\right)}}$	1513.683	874.490	450.189
${\overset{¯}{y}}_{6_{\left(0.35\right)}}$	1704.629	984.804	506.979
${\overset{¯}{y}}_{7_{\left(0.35\right)}}$	4796.254	2770.909	1426.471
${\overset{¯}{y}}_{8_{\left(0.35\right)}}$	3022.194	1745.993	898.841
${\overset{¯}{y}}_{9_{\left(0.35\right)}}$	3557.394	2055.191	1058.017

The outcomes of the numerical investigation are presented in Table 2, Table 3, Table 4, Table 5, Table 6, Table 7 for both sensitive and non-sensitive scenarios. Upon examining Table 2, Table 3, Table 4, Table 5, Table 6, Table 7, it becomes evident that each estimator $\left({\overset{¯}{y}}_{t_{\left(q\right)}},{\overset{¯}{z}}_{t_{\left(q\right)}}\right)$, with q=(0.15,0.25,0.35), within the proposed category outperforms its counterpart in the existing category. Consequently, in both instances, the new estimators exhibit a notable proficiency improvement across populations 1-3.

## 6 Conclusion

In this manuscript, building upon recent advancements in design-based sampling, we introduce a resilient group of quantile regression based mean estimators. These estimators demonstrate superior performance compared to several existing methods when confronted with extreme observations. The proposed class not only incorporates the mean of sampled observations but also considers the mean of unsampled observations of *X*. In a typical scenario where the gathered values of *Y* variable are not influenced by socially desirable bias, we formulate the expression for the minimum MSE. Furthermore, we have expanded the utilization of the suggested class to situations where the study variable is deemed sensitive. The values are collected from survey units through scrambled responses, with the intention of preserving respondent privacy, promoting cooperation, and addressing nonresponse rates or inaccurate answers. In this context, we explore an additive scrambled response model to perturb the provided responses. To ensure brevity, we have conducted theoretical computations in conjunction with numerical experiments using different data sets. These computations distinctly highlight the superior performance of the new class in both nonsensitive and sensitive settings, at least as illustrated within the experimental contexts outlined in the manuscript. Consequently, survey practitioners may find the proposed class particularly valuable, as it holds the potential to enhance the likelihood of obtaining more efficient estimates for the parameter of interest. In future studies, the work can be extended under systematic sampling.

## CRediT authorship contribution statement

**Abdullah Mohammed Alomair:** Writing – review & editing, Writing – original draft, Visualization, Validation, Supervision, Software, Resources, Project administration, Methodology, Investigation, Funding acquisition, Formal analysis, Data curation, Conceptualization. **Soofia Iftikhar:** Writing – review & editing, Writing – original draft, Visualization, Resources, Data curation, Conceptualization.

## Declaration of Competing Interest

The authors declare that they have no known competing financial interests or personal relationships that could have appeared to influence the work reported in this paper.

## Data Availability

Data will be made available on request.
